# Higher temperatures reduce the number of *Trypanosoma cruzi* parasites in the vector *Triatoma pallidipennis*

**DOI:** 10.1186/s13071-021-04872-6

**Published:** 2021-08-04

**Authors:** Berenice González-Rete, Ana E. Gutiérrez-Cabrera, José Antonio de Fuentes-Vicente, Paz María Salazar-Schettino, Margarita Cabrera-Bravo, Alex Córdoba-Aguilar

**Affiliations:** 1 Posgrado en Ciencias Biológicas, Universidad Nacional Autónoma de México, Ciudad de México, Mexico; 2 Departamento de Microbiología Y Parasitología, Facultad de Medicina, Universidad Nacional Autónoma de México, Ciudad de México, Mexico; 3 CONACYT-Centro de Investigación Sobre Enfermedades Infecciosas, Instituto Nacional de Salud Pública, Cuernavaca, Morelos Mexico; 4 Instituto de Ciencias Biológicas, Universidad de Ciencias Y Artes de Chiapas, Tuxtla Gutiérrez, Chiapas Mexico; 5 Departamento de Ecología Evolutiva, Instituto de Ecología, Universidad Nacional Autónoma de México, Coyoacán, Ciudad de México, Mexico

**Keywords:** *Triatoma pallidipennis*, *Trypanosoma cruzi*, Strains, Global warming, Temperature, Parasites

## Abstract

**Background:**

Relatively little is known about how pathogens transmitted by vector insects are affected by changing temperatures analogous to those occurring in the present global warming scenario. One expectation is that, like their ectothermic vectors, an increase in temperature could reduce their fitness. Here, we have investigated the effect of high temperatures on the abundance of *Trypanosoma cruzi* parasites during infection in the vector *Triatoma pallidipennis*.

**Methods:**

We exposed *T. pallidipennis* nymphs to two strains (Morelos and Chilpancingo) of *T. cruzi*. Once infected, the fifth-instar bugs were distributed among three different temperature groups, i.e. 20, 30, and 34 °C, and the resulting parasites were counted when the bugs reached adulthood.

**Results:**

The number of parasites increased linearly with time at 20 °C and, to a lesser extent, at 30 °C, especially in the Chilpancingo compared to the Morelos strain. Conversely, at 34 °C, the number of parasites of both strains decreased significantly compared to the other two temperatures.

**Conclusions:**

These results suggest negative effects on the abundance of *T. cruzi* in *T. pallidipennis* at high temperatures. This is the first evidence of the effect of high temperatures on a pathogenic agent transmitted by an insect vector in the context of global warming. Further tests should be done to determine whether this pattern occurs with other triatomine species and *T. cruzi* strains.

**Graphical abstract:**

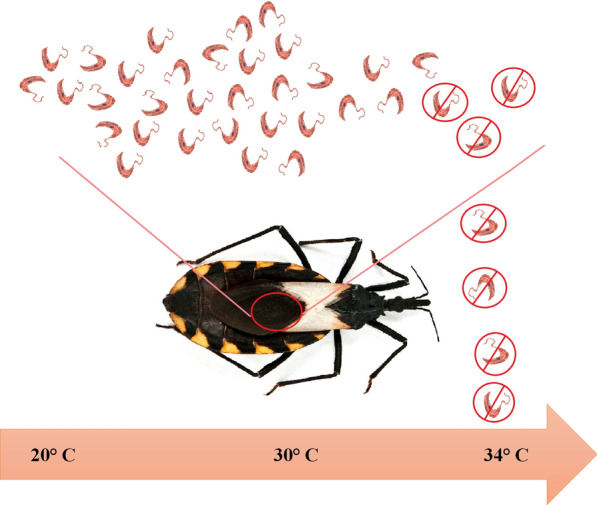

## Background

Against the backdrop of an ever-warmer climate estimated by the Intergovernmental Panel on Climate Change (IPCC) [1, 2], one of the most urgent questions involves the behavior of the major threats to humanity. One such threat is vector-borne diseases. These diseases are zoonoses in which an agent, generally an arthropod, acts as a stopover for pathogens that ultimately infect humans. The most well-known example is the mosquito, which works as a vehicle for several pathogenic agents, including parasites, bacteria, and viruses. Knowledge of the extent that these infectious agents will be affected by global warming is key to understanding the future of zoonoses [3].

The number of people infected by vector-borne pathogens is generally on the rise [3, 4], facilitated largely by global temperature increases [5]. These conclusions are based on a review of the literature, which has documented vector-borne disease in zones around the world in which they had not been previously found, and the increased number of cases of some diseases in their endemic zones [6]. However, this type of analysis does not provide details on the particular mechanisms of zoonosis that will be affected by temperature increases [4, 7]. The mechanical parameters that will be affected are difficult to estimate precisely, so empirical models are more feasible [6]. For example, a series of disparate phenomena will favor vector-borne diseases, from land-use change [8, 9], human migration [10] and urbanization [3], to the rate of insect development [11, 12], to name just a few. As such, the multifactorial nature of vector-borne diseases requires data on each of the factors that favor the increase in transmission of those diseases, in order to assemble different parts of the puzzle and understand the main causes [7].

One of the least studied factors of zoonosis in the context of high temperatures is the vector–pathogen relationship. Given that vectors are ectotherms and that pathogens develop within them, high temperatures are expected to generate, for example, similar responses in both, although this is not always true [13]. Although the development of the vector can be accelerated in some cases [14, 15], this is not always the case [16]. On one hand, this is understood because arthropods can increase their fitness up to a point, after which there will be viability costs [17–19]. On the other hand, the temperature ranges that maximize fitness vary among vector species [20]. Meanwhile, pathogens may not react to temperature changes in the same way as their vectors [21]. In fact, the thermal optima of pathogens and vectors are rarely the same, with the optima of pathogens varying more as a function of their distribution areas (e.g., temperate or tropical) than their vectors [21]. Therefore, we know relatively little about how ambient temperatures, especially high temperatures, affect the fitness of vector-borne pathogens.

Chagas disease is one disease whose risk is expected to increase in the future [22]. This disease is caused by the protozoan *Trypanosoma cruzi*, which uses the triatomine bugs (Hemiptera: Reduviidae) as vectors, and a variety of vertebrates, including humans, as hosts [23, 24]. In the case of humans, the parasite causes Chagas disease, an affliction that can lead mainly to heart and digestive problems. The bugs feed on blood that contains the parasite, and the parasite reproduces exponentially inside the bug within a few weeks [24]. There are few studies on how high temperatures affect the bug-*T. cruzi* pair. The most robust evidence of the effect of high temperatures comes from a recent study on the bug *Rhodnius prolixus* and *T. cruzi* [25]. This study suggests that the bugs accelerate their development and fecundity while the parasites multiply [25]. However, the high temperature (30 °C) chosen does not necessarily simulate what the bugs would experience under global warming [25]. A second study exposed the bug *Triatoma pallidipennis* infected with *T. cruzi* to 34 °C, a temperature similar to what is expected under global warming. These authors found that the bugs died faster at 34 °C than at relatively low temperatures [26]. However, although it used infected bugs, the study did not measure the effect of the high temperatures on the fitness of the parasite.

In this study, we have investigated the effect of high temperatures on the abundance of *T. cruzi* parasites during infection in the vector *T. pallidipennis*. To do this, we used two strains of the parasite, Morelos and Chilpancingo, for two important reasons. First, previous studies have shown that these strains induce different behavioral changes [27] and fitness costs [28] in *T. pallidipennis*. Second, *T. cruzi* is divided into several groups and strains, which may be adapted to certain ambient temperatures [29]. In fact, the development of the different strains is sensitive to temperature in the laboratory [30]. This suggests that the different strains may vary in their fitness response to different temperatures. Given that the same species of triatomine can carry different strains of *T. cruzi* depending on the geographic area where the bug is found [23], the different strains could experience different temperatures. Thus, using Morelos and Chilpancingo allows us to understand more generally, and perhaps more realistically, the possible negative effects of high temperatures on parasites.

## Methods

### Individuals of *T. pallidipennis*

We used nymphs that had recently molted into the fifth instar from an insectarium (28 °C and 60% relative humidity, 12/12 h light/dark cycle) from the Faculty of Medicine at the Universidad Nacional Autónoma de México (Mexico City). Bugs were chosen randomly for all experiments.

### *Trypanosoma cruzi* isolates

The first strain was ITRI/MX/12/MOR (hereafter, Morelos), which was obtained from a male of *T. pallidipennis* captured and isolated in 2012 in Cuernavaca, Morelos, Mexico. The other strain was ITRI/MX/14/CHIL (hereafter, Chilpancingo), which was obtained from a *T. pallidipennis* female captured and isolated in 2014 in Chilpancingo, Guerrero, Mexico. Both strains belong to the genetic group TcI [28] and were maintained in female CD-1 mice (15–18 g).

### Incubation temperatures

Considering the temperature range at which *T. pallidipennis* infected with *T. cruzi* have been reported to survive, three previously studied temperatures were chosen: a minimum temperature (20 °C), a preferred temperature for the development of the parasite and vector (30 °C) [31], and a maximum temperature under the climate change panorama (34 °C) according to scenarios of the IPCC [1, 2] and previous experimental studies of the responses of *T. pallidipennis* [26]. These temperatures were set in incubators with constant airflow, 60% relative humidity, and a photoperiod of 12:12 h light/dark (FE-131AD, FELISA, Mexico City).

### Feeding of *T. pallidipennis* nymphs

Three hundred and sixty fifth-instar nymphs were infected by feeding on the two *T. cruzi* strains from female CD-1 mice (15–18 g) that had been previously inoculated with a concentration of 20,000 blood trypomastigotes/ml [32]: 180 nymphs were fed mice infected with the Morelos strain (Morelos group) and 180 with the Chilpancingo strain (Chilpancingo group). The mice were used 15 days post-infection, corresponding to the exponential reproduction phase of *T. cruzi* [32]. The nymphs were fed for 15–20 min in separate groups of five nymphs per infected mouse in the dark until they showed clear signs of considerable weight increase (distension of the abdomen to approximately double its thickness before feeding). With respect to the level of parasitemia in the mice, each of the infected nymphs ingested an average of 8000 parasites.

### Incubation of insects

After feeding and infection, the nymphs were placed in plastic jars (one per jar) and labeled for their identification. One hundred and twenty nymphs were incubated at 20 ± 2 °C, of which 60 were infected with the Morelos strain and 60 with the Chilpancingo strain. Similarly, 120 nymphs were incubated at 30 ± 2 °C and 120 nymphs at 34 ± 2 °C. The incubation period was from 5 to 60 days after feeding, with groups of nymphs incubated different times for 5, 10, 15, 20, 25, 30, 35, 40, 45, 50, 55, and 60 days for each strain. The bugs were distributed at random. Animals of all three groups and respective temperatures were fed every 5 days as indicated above using non-infected mice.

### Confirmation of infection

After the incubation time of the nymphs (5 to 60 days), the rectal contents of each nymph were examined by abdominal compression, to confirm the presence of infection by *T. cruzi* by direct observation [32]. We placed a drop of feces dissolved in phosphate-buffered saline (PBS) 1X, pH 7.2, on a microscope slide, taking 10 μl, which was observed under the microscope at a magnification of ×40 (Olympus CH-2, Center Valley, PA, USA) [32].

### Quantification of the total parasites in the rectum

After confirming the infection, the insects were dissected under a stereoscopic microscope (Stemi 2000, Carl Zeiss, Jena, Germany 426126). The extremities were removed with dissection forceps and the abdomen was disinfected with 70% alcohol. The connexivum was identified and cut to expose the abdominal cavity, and the Malpighian tubules and fat body were removed [32]. The rectum of each nymph was identified and placed separately in a 0.6 ml Eppendorf tube with 100 µl of sterile PBS 1X (pH 7.2), where the rectal contents were homogenized.

In a Neubauer chamber we counted the total parasites present in the rectal ampulla of each of the nymphs using 10 μl of the homogenate described above. The quantification was done in duplicate, using the following formula (Eq. 1):

1 $${\text{Total}}\;\# \;{\text{of}}\;{\text{parasites}} = \frac{{\# \;{\text{of}}\;{\text{parasites }}\left( {4\;{\text{quadrants}}} \right) \times {\text{dilutionfactor}}}}{4}\left( {{\text{final}}\;{\text{volume}}} \right)$$

The number of parasites present in four quadrants was multiplied by the dilution factor (10,000) then divided by 4, to obtain the total number of parasites in the final volume (100 μl).

### Statistical analysis

We used the Kolmogorov–Smirnov (K–S) test to determine normality and homogeneity of variance. Once we confirmed the normal distribution of the data, we tested whether the number of parasites present in the rectal ampulla of the nymphs differed among all treatments using a univariate general linear model. In this statistical model, the total number of parasites was set as the dependent variable, while as predictor variables we set (1) the two parasite strains (Morelos and Chilpancingo parasites), (2) the three different temperatures (20 °C, 30 °C, and 34 °C), and (3) the 12 different incubation times (5 to 60 days). Our objective was to determine whether both the model as a whole and each of the predictor variables separately, as well as the interaction among the three predictor variables, were significant. This model would allow us to see whether each variable or the interaction of variables predicted parasite numbers. In the case of the interaction, we compared the groups using 95% confidence intervals. This analysis was done using SPSS version 24.0 software. All data are expressed as the average number of parasites ± standard error.

## Results

**Fig. 1:**
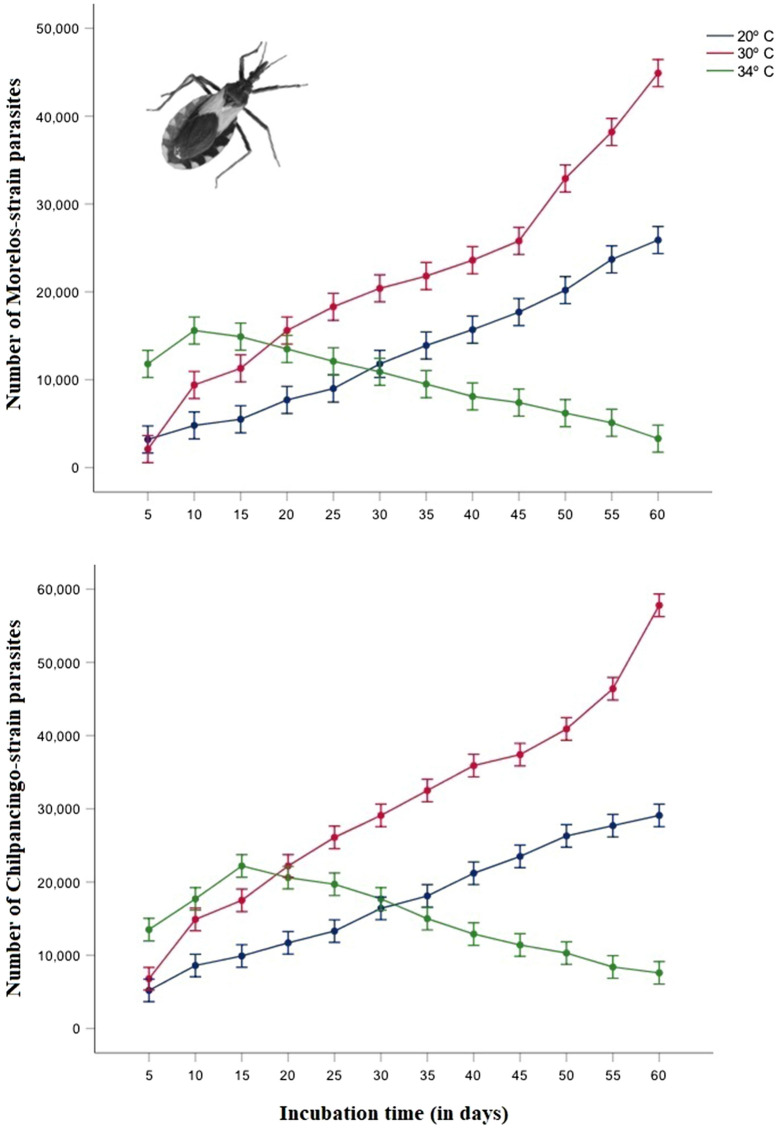
Total number of parasites in the rectal ampulla of fifth-instar nymphs of *Triatoma. pallidipennis* infected with the Morelos (upper) and Chilpancingo (lower) strains of *Trypanosoma cruzi*. The numbers indicate mean ± standard error

The model indicated that there were significant differences depending on the parasite strain (Morelos and Chilpancingo), temperature (20 °C, 30 °C, and 34 °C), and incubation time (5, 10, 15, 20, 25, 30, 35, 40, 45, 50, 55, and 60 days; Table 1). All interactions among these three predictor variables were also significant (Table 1). These results indicate that as the temperature increased (20 °C to 30 °C), the number of parasites from the Morelos and Chilpancingo strains increased, although these differences disappeared at the highest temperature (34 °C) (Fig. 1). However, at day 5, there were more parasites when they were kept at 34 °C.

**Table 1:** Results of the univariate general linear model of the total number of *Trypanosoma cruzi* parasites (Chilpancingo or Morelos strains) in the rectum of *Triatoma pallidipennis*, according to the infection status, temperature (20, 30, and 34 °C), and incubation time (5, 10, 15, 20, 25, 30, 35, 40, 45, 50, 55, and 60 days), and their interactions

Terms	Type III sum of squares	d*f*	Mean square	*F*	*P*
Corrected model	44,813,532,638.889	71	631,176,516.041	205.725	0.0001
Intercept	116,874,117,361.113	1	116,874,117,361.113	38,093.873	0.0001
Infection status	3,171,367,361.111	1	3,171,367,361.111	1033.673	0.0001
Temperature	13,002,218,055.556	2	6,501,109,027.778	2118.967	0.0001
Incubation time	11,068,524,305.556	11	1,006,229,482.323	327.970	0.0001
Status * temperature	324,009,722.222	2	162,004,861.111	52.804	0.0001
Status * time	161,007,638.889	11	14,637,058.081	4.771	0.0001
Temperature * time	16,898,365,277.778	22	768,107,512.626	250.356	0.0001
Status * temperature * time	188,040,277.778	22	8,547,285.354	2.786	0.0001
Error	883,600,000.000	288	0.355		
Total	162,571,250,000.000	360			
Adjusted total	45,697,132,638.889	359			

*P* values of each corresponding *F* value indicate whether each predictor is statistically significant

The status of infection affected the total number of parasites, with the Morelos strain having a lower number of parasites in the rectum at all three temperatures compared to the Chilpancingo strain (Fig. 1). The incubation time was also a good predictor, where over time there was an increase in the number of parasites in both the Morelos and Chilpancingo strains (20 °C to 30 °C). This effect was inverted, however, at 34 °C, in which the Morelos strain had its peak number of parasites at 10 days and the Chilpancingo strain had its peak at 15 days (Fig. 1).

## Discussion

Temperatures from 20° to 34 °C initially led to positive effects on the number of parasites in the rectum. This positive effect was clear at the onset of the experiment for a temperature of 34 °C but soon declined at 10 to 15 days, while temperatures of 20 and 30 °C led to a steady increase. Ideally, additional temperatures between 30° and 34 °C would have allowed us to determine exactly where the decline took place. However, since we used only parasites from the rectal ampulla, more parasites may be present in other intestinal regions. In any case, one explanation is that, as occurs in ectotherms, high temperatures lead to physiological compromises, likely derived from the conditions that the host experiences. For example, there is evidence that in fungus–insect relationships, the viability of the former is highly dependent on the external environment of the host (for a specific case see [33]; for a review see [34]). This reduced viability of the parasite could be interpreted as reduced virulence above a temperature threshold [34]. This non-linear relationship of virulence, estimated as parasite abundance in this case, is typical of parasite–host relationships [35, 36]. It is therefore likely that the replicative capacity of the parasite decreases at high temperatures. Another explanation is that, as a consequence of the temperature increase, the metabolism of the bug accelerates and the energy resources are depleted in a shorter time.

One question related to reduced virulence is whether the negative effects also occur in the host bug. Related to this, *T. pallidipennis* bugs infected with the same strains also experienced decreased survival and immune response at 34 °C compared to infected bugs kept at 20° and 30 °C [26]. Therefore, the reduction in the fitness of parasites and hosts and defense in hosts at high temperatures may be because neither of the two agents is tolerant to too-warm environments. This result contrasts, however, with previous ideas on different responses to temperature in parasites and insect vectors based on spatial scales and geographic mosaics [13]. Our present results also contrast with another triatomine vector, *R. prolixus*. In this study, *R. prolixus*-infected bugs were exposed to three different temperatures—26 °C, 28 °C, and 30 °C—and the number of parasites was analyzed [25]. In general, a temperature of 30 °C gave rise to more parasites, rather than fewer, as occurred in our study. The study probably did not involve a thermal regime above a temperature threshold [25]. In this sense, our study clearly establishes a thermal limit for both strains, which together with the other study indicates that above that threshold, the vectorial efficiency of *T. pallidipennis* decreases [26].

The differences in the growth of the two strains of the parasite at the three temperatures may reflect the coadaptation of these strains with their host, but with different expression from the perspective of an evolutionary arms race. One way of understanding this arms race can be found in a study that utilized the same species of bug infected with the two strains of parasite that we used, and found different fitness costs to the bug depending on the strain [28]. For example, the Chilpancingo strain reduced the size and survival of the bugs more than the Morelos strain [28]. Consistent with this effect, the Chilpancingo strain produced more parasites within the bug than the Morelos strain [28]. Our results corroborate that at the three temperatures, the Chilpancingo strain gave rise to more parasites. According to a coevolutionary arms race scenario, and independent of temperature, the arms race favors the Chilpancingo strain. This differential cost according to strain remains even at high temperatures [26].

As far as we know, our study is the first to use different temperatures to generate fitness trajectories and demonstrate that some strains can vary in virulence. To what extent can this be interpreted in a geographic context? Indeed, the geographic mosaic of how the different strains of the parasite are distributed indicates that some groups can predominate over others in certain zones [37–39]. For example, the groups TcI to TcIV are clearly differentiated in distribution from Mexico to Colombia from data gathered from *T. dimidiata* bugs [40]. Interestingly, the different groups appear to be adapted to different ambient temperatures. For example, group TcV was more likely to be found in people infected in zones with higher annual temperatures than group TcVI [29]. According to our results, these differences could be due to the greater ability of some groups to tolerate certain temperatures. It would be interesting to collect different bug species at different temperatures and compare the strains or groups of *T. cruzi* to see whether they are consistent with our results. Ideally, both hosts and strains should also be examined using fluctuating diurnal temperatures, as these can have different impacts on vector competence, as shown for mosquitoes and dengue virus [41, 42].

Previous studies that used temperature to predict parasite–host geographical occurrence have been based on niche models that hypothesize the future changes in the distribution areas of both agents [43]. In the case of triatomine bugs, these models have provided evidence of an increase in distribution towards the temperate zones of North America [44], although these models do not consider the interaction with *T. cruzi*, which could affect the model precision [45]. In any case, our findings need to be tested using other important triatomine species and parasite strains to complete our still unclear panorama of parasite–host geographical occurrence and, thus, risk of Chagas disease.

## Conclusions

Our results suggest negative effects on the abundance of *T. cruzi* in *T. pallidipennis* at high temperatures. These results indicate that increased temperatures could reduce the vectorial capacity in at least one triatomine species and two parasite strains. These results are opposite to those of non-experimental studies that have used environmental variables to envisage future scenarios, and call our attention to the need for more experimental studies.

## Data Availability

The datasets used and/or analyzed during the present study are available from the corresponding author upon request.

## Abbreviation

IPCC: Intergovernmental Panel on Climate Change
